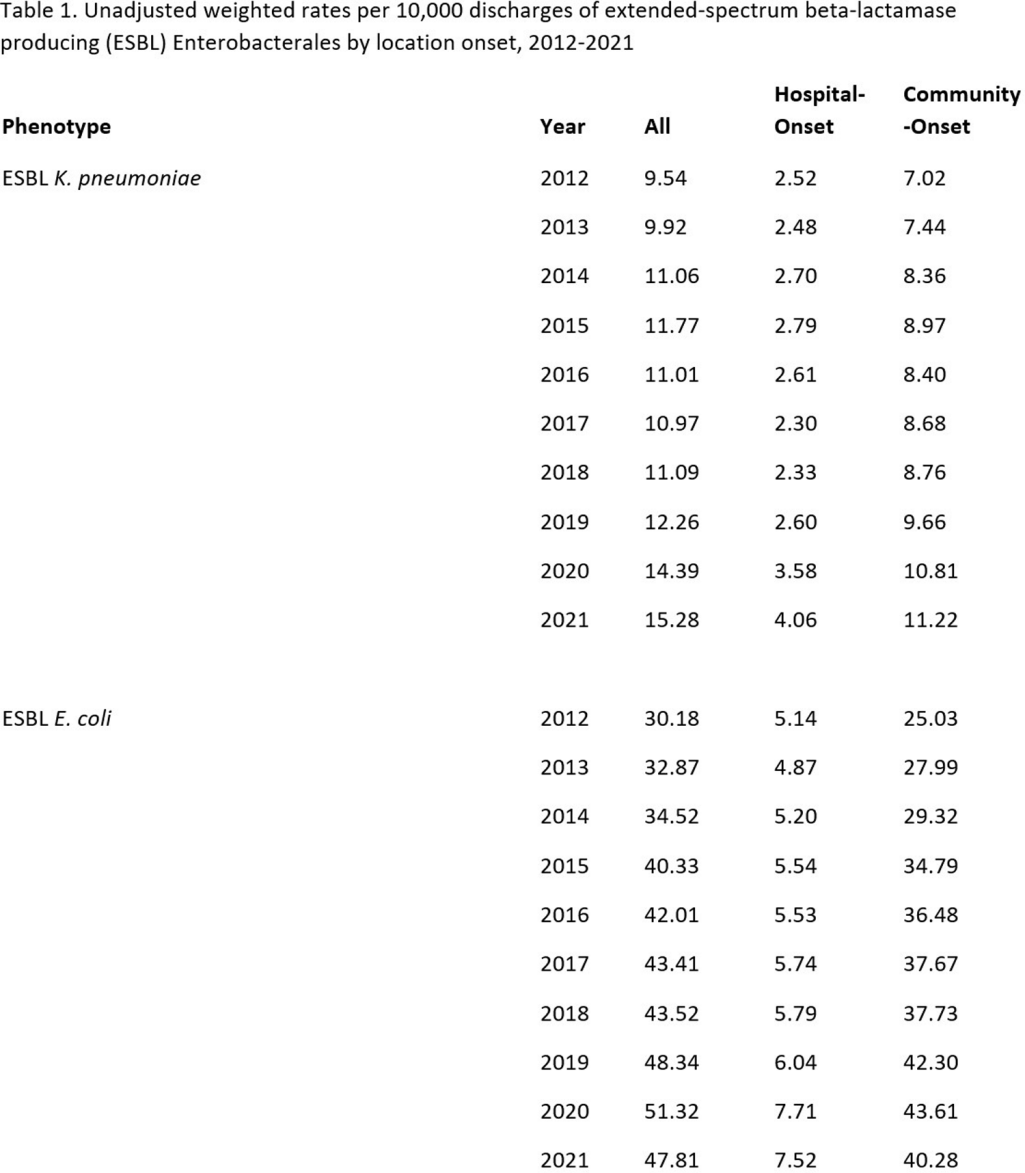# Extended-Spectrum Beta-Lactamase Producing Enterobacterales Infections in the United States, 2012-2021

**DOI:** 10.1017/ash.2024.141

**Published:** 2024-09-16

**Authors:** Alexander Maillis, Natalie McCarthy, Hannah Wolford, James Baggs, Sujan Reddy, Joseph Lutgring

**Affiliations:** Centers for Disease Control and Prevention; Chenega

## Abstract

**Background:** The 2022 Special Report: COVID-19 U.S. Impact on Antimicrobial Resistance identified continued increases in the rate of extended- spectrum beta-lactamase producing (ESBL) infections in the United States from 2017 through 2020. Using similar data sources and methodology, we examined the trends of species-specific ESBL infections from 2012-2021. **Methods:** We identified a cohort of patients from the PINC AI and BD Research Insights databases with a clinical culture yielding a Klebsiella pneumoniae or Escherichia coli isolate with accompanying susceptibility testing. E. coli or K. pneumoniae isolates non-susceptible to ceftriaxone, cefotaxime, ceftazidime, or cefepime were considered suggestive of ESBL production. Isolates from patients with no culture yielding the same resistance phenotype of interest in the previous 14 days were counted as an incident case. Community-onset (CO) cultures were obtained ≤ day 3 of hospitalization; hospital-onset (HO) cultures were obtained ≥ day 4. We used a raking procedure to determine weights for extrapolating the number of discharges included in our sample to match the distribution of discharges, stratified by bed size, U.S. census division, urban/rural designation, and teaching status, for U.S. hospitals included in the American Hospital Association survey. We evaluated rates over time due to the changes in number of hospitalizations during the COVID-19 pandemic. Results were stratified by HO and CO, and sterile and non-sterile specimen sources. **Results:** In 2021, there were 48,936 ESBL K. pneumoniae and 153,112 ESBL E. coli infections among approximately 32 million discharges. Overall, most infections were CO and from non-sterile specimens. From 2012-2021, the rate of ESBL K. pneumoniae increased from 9.54 to 15.28 per 10,000 discharges. ESBL E. coli infections increased from 2012-2020 (30.18 to 51.32 per 10,000 discharges), then declined in 2021 (47.81 per 10,000 discharges) (Table 1, Figure 1). The proportion of non-sterile ESBL E. coli declined from 88% in 2012 to 83% in 2021, and the proportion of non-sterile ESBL K. pneumoniae was 85-87% over the study period (Figure 2). **Conclusion:** ESBL E. coli and K. pneumoniae infections increased from 2012-2021, although the CO ESBL E. coli rate decreased between 2020 and 2021. Understanding changes in culturing practices over time may provide insights into the increased proportion of ESBL E. coli from sterile sites. Additionally, further investigation into differences in organism trends, particularly in 2021, may inform prevention strategies.

**Figure:**
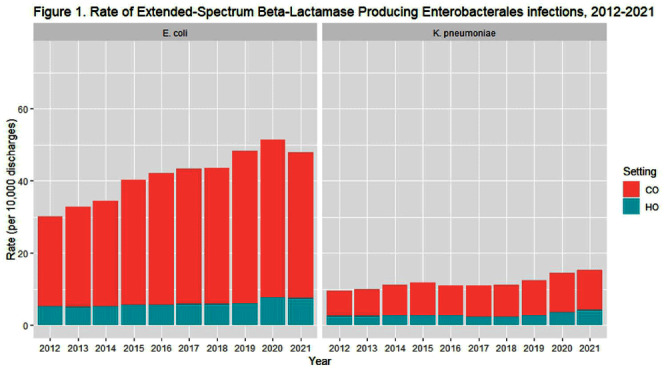

**Figure:**
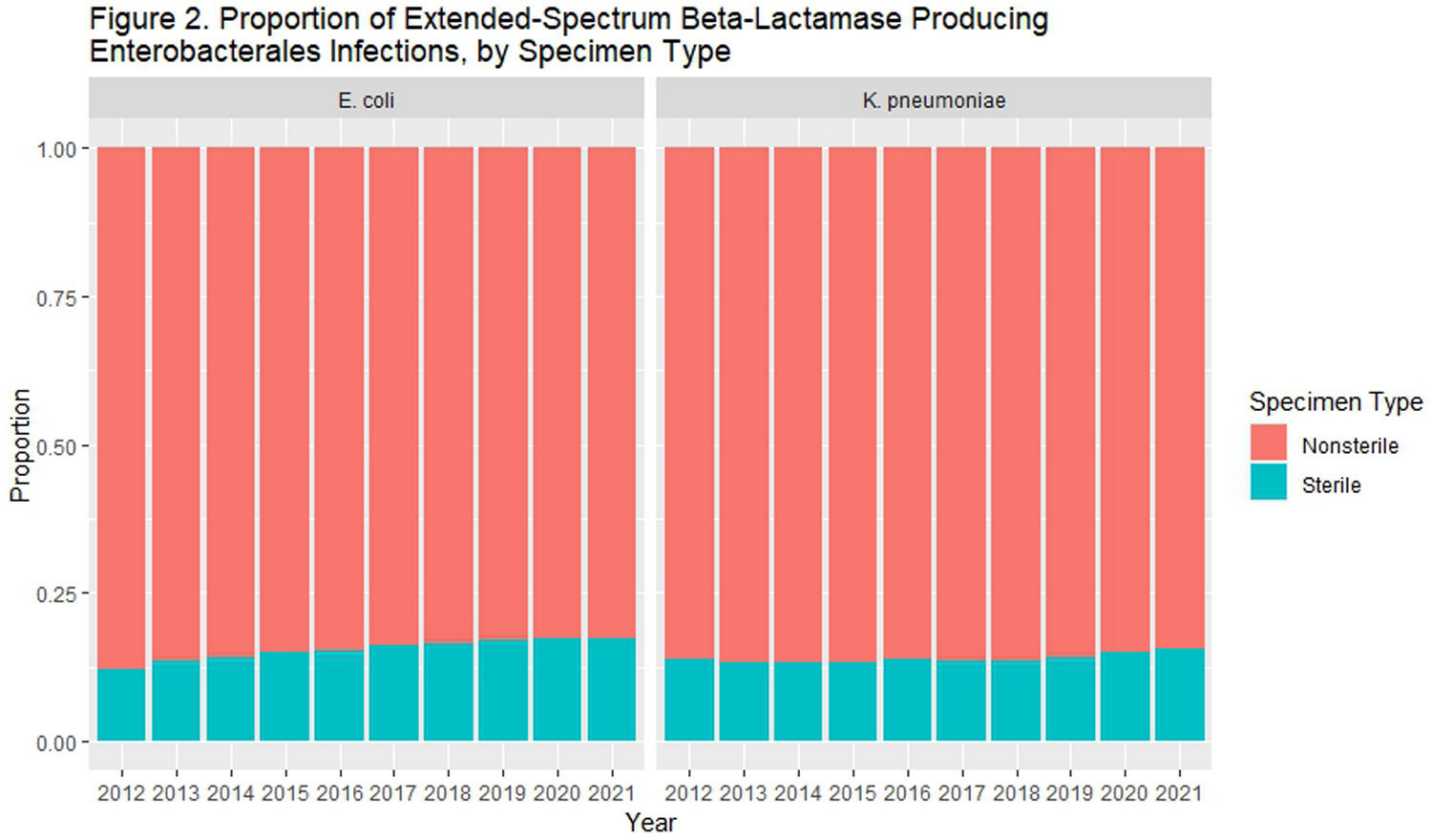

**Figure:**